# Consequences of changing rainfall for fungal pathogen-induced mortality in tropical tree seedlings

**DOI:** 10.1002/ece3.252

**Published:** 2012-07

**Authors:** Tom Swinfield, Owen T Lewis, Robert Bagchi, Robert P Freckleton

**Affiliations:** 1 Department of Animal and Plant Science, University of SheffieldSheffield, S10 2TN, United Kingdom; 2 Department of Zoology, University of OxfordOxford, OX1 3PS, United Kingdom

**Keywords:** Climate change, Janzen–Connell, pathogens, rainfall, species coexistence

## Abstract

Most general circulation models predict that most tropical forests will experience lower and less frequent rainfall in future as a result of climate change, which may reduce the capacity of fungal pathogens to drive density-dependent tree mortality. This is potentially significant because fungal pathogens are thought to play a key role in promoting and structuring plant diversity in tropical forests through the Janzen–Connell mechanism. Therefore, we hypothesize that the drying of tropical forests will negatively impact species coexistence. To test one prediction of this hypothesis, we imposed experimental watering regimes on the seedlings of a tropical tree, *Pleradenophora longicuspis*, and measured mortality induced by fungal pathogens under shade house conditions. The frequency of watering had a strong impact on survival. Seedlings watered daily experienced significantly higher mortality than those watered every three or every six days, while increasing the volume of water applied also led to increased mortality, although this relationship was less pronounced. These results suggest that the capacity of fungal pathogens to drive density-dependent mortality may be reduced in drier climates and when rainfall is less frequent, with potential implications for the diversity enhancing Janzen–Connell mechanism.

## Introduction

Natural enemies are thought to play a major role in maintaining the structure and diversity of tropical forests. Herbivores, seed predators, and fungal pathogens inhibit the recruitment of new conspecific individuals in the immediate vicinity of adult trees (Janzen 1970; Connell 1971), and promote the recruitment of heterospecifics (Freckleton and Lewis 2006), the so-called Janzen–Connell mechanism. Fungal pathogens have received considerable attention as key drivers of this process because they can reduce survival to almost nil when seedlings occur at the highest densities (Bagchi et al. 2010), while the survival of heterospecifics appears to be relatively unaffected (Mangan et al. 2010). However, abiotic factors such as humidity, precipitation, and temperature are likely to affect fungal pathogen virulence (the reduction in host fitness) and transmission (the potential for pathogens to move between hosts) (Dorrance et al. 2003). Negative effects on plant growth and mortality imposed by fungal pathogens are therefore expected to vary with climate, altering the strength of Janzen–Connell effects, and ultimately affecting local species richness.

Over the next century substantial climate change has been forecast, with most tropical forests predicted to become warmer and drier (Christensen et al. 2007; Malhi et al. 2008; Corlett 2011). Under these changed conditions, it seems likely that the virulence of fungal pathogens will be altered (Burdon et al. 2006; Thompson et al. 2010) but there is very little empirical evidence to suggest in which direction. Increased temperature may increase the metabolic rates of fungal pathogens and increase infection (Thompson et al. 2010). Alternatively, seed and seedling mortality from fungal pathogens may be reduced under lower rainfall regimes (Givnish 1999) because moisture may be required to induce sporulation and promote dispersal, while drought-induced stomatal closure may prevent foliar fungal pathogens from penetrating plant tissues (Garrett et al. 2006). However, we are unaware of experiments that attempt to measure the effects of altered abiotic conditions on the transmission and virulence of fungal pathogens in tropical forests.

**Figure 1:**
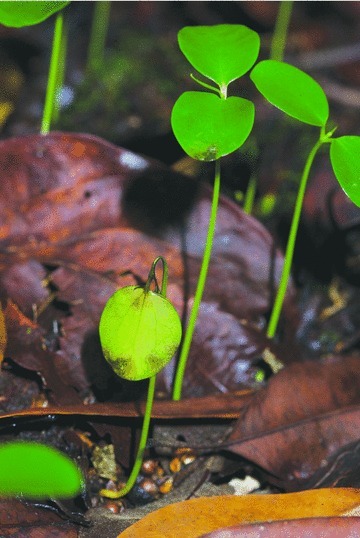
*Pleradenophora longicuspis* seedlings succumbing to fungal pathogen-induced mortality.

In this study, we assessed the potential for fungal pathogens to drive seedling mortality under a variety of artificial watering regimes in a simple manipulative experiment under seminatural shade house conditions. The seedlings of the canopy tree *Pleradenophora longicuspis* (Standl.) Esser (Euphorbiaceae) were exposed to a gradient of watering volumes at three different frequencies, to test the prediction that mortality driven by fungal pathogens increases with both the volume and frequency of watering (Fig. 1). To assess the role of foliar and edaphic moisture in determining the activity of these fungal pathogens, water was applied either from above seedlings, ensuring that both soil and foliage became wet, or directly to the soil, avoiding wetting the foliage. A fungicide was also applied to measure seedling survival in the absence of fungal pathogens.

## Material and Methods

### Study system

The experiment was conducted during July and August 2009 in a screened, rainproof shade house in the forest close to the Las Cuevas Research Station, within the Chiquibul Forest Reserve, Belize. *Pleradenophora longicuspis* (Standl.; Euphorbiaceae) is an abundant pioneer tree at this location and produces balistically dispersed seeds that germinate rapidly to form high-density seedling carpets (up to 1650 seedlings m^−2^; Bagchi et al. 2010). Fungal pathogens have been shown to drive density-dependent seedling mortality in this species (Bell et al. 2006), so that almost no seedlings survive at the highest initial densities (Bagchi et al. 2010). Note that Bell et al. (2006) incorrectly refer to *P. longicuspis* as “*Sebastiana longicuspis*.” In separate studies at this field site, strains of *Colletotrichum* sp. and *Diaporthe* sp. have been implicated in causing mortality in young seedlings of *P. longicuspis* (R. Gallery et al.*,* unpubl. data).

The top approximately 10 cm of soil and humic matter was gathered from four locations where *P. longicuspis* grows at high density. These samples were combined and mixed thoroughly before being passed through a 1 × 1 cm mesh screen to remove large stones, roots, and seeds. Seventy-eight seed trays (12 × 12 × 5 cm), without drainage holes, were filled with 400 mL of processed soil, which had been air-dried for approximately one month. Healthy two-day-old seedlings were gathered from beneath three areas of high *P. longicuspis* tree density and 20 seedlings, selected at random, were transplanted into each tray. This generated a density equivalent to approximately 1388 seedlings m^−2^, comparable to the highest densities typically observed in the field, at which density-dependent mortality is usually very high (Bagchi et al. 2010).

### Simulated rainfall regimes

Immediately prior to seedling transplantation, 150 mL of water was added to each tray; all water used in this experiment was collected from an underground aquifer. Seedlings were then given two days to establish before simulated rainfall regimes commenced. Regimes were randomly assigned to trays, which were arranged within the shade house, separated by at least 20 cm. Simulated rainfall was applied as a directed spray either (1) from above, wetting both the foliage and the soil, or (2) directly to the soil surface, with special effort made to avoid wetting the leaves. The “leaf wetting” method was intended to reflect natural rainfall, while the “soil wetting” method provided a comparison in which foliar moisture remained at consistently low levels, enabling the mechanism for transmission to be detected. Each of these watering methods was replicated in 30 trays, while an additional 18 trays were treated with the fungicides Ridomil Gold® and Amistar® applied, in combination, in accordance with the manufacturer's guidelines: 0.25 g m^−2^ of Ridomil® and 0.005 g m^−2^ of Amistar®, each dissolved in 100 mL m^−2^.

One-third of the trays, within each of the watering methodologies (leaf wetting, soil wetting, and fungicide), received simulated rainfall at each of three different frequencies: daily, every three days, or every six days. In this way, watering method × frequency combination was replicated in 10 trays, within both the leaf wetting and soil wetting methodologies, but only six trays within the fungicide method. Within each method × frequency combination each tray received a different total volume of simulated rainfall (during each six-day period), so that a gradient in the volume of simulated rainfall was established. The smallest and largest volumes applied were set to 20 mL and 200 mL, respectively, per six-day simulated rainfall period, with intermediate volumes attributed at 20 mL intervals. For the fungicide method, a subset of these volumes (20, 40, 80, 120, 160, and 200 mL) was used. The precise volume of water applied to each tray at each rainfall event was determined by dividing this total volume by the frequency of rainfall application it received during the six-day period. In this way, there was no replication for each individual method × frequency × volume combination. Fungicides were applied at the onset of the simulated rainfall regimes and every sixth day thereafter. When fungicides were applied a volume of water equivalent to that used to dissolve the fungicides (3 mL) was subtracted from the volume applied on that day. A soil-wetting fungicide was not used because the fungicides have a systemic action and are designed to be applied as a foliar spray; applying to the soil may have rendered them ineffective. Seedling survival was recorded approximately every five days between the 8 and 30 August 2009. Previous work on this system suggests that this is sufficient time to document the peak period of mortality driven by fungal pathogens (Bagchi et al. 2010).

### Statistical analysis

The probability of seedling survival for each replicate was calculated as the proportion of successfully transplanted seedlings (those surviving after the initial establishment period) surviving to the end of the experiment. The data were analyzed using generalized linear models (GLMs), with a quasibinomial error structure to correct for overdispersion, using the statistical software package R (version 2.12.2). Seedling survival was modeled as a function of the volume of simulated rainfall (continuous variable), frequency (factor with three levels), and method (factor with three levels). In addition, survival was modeled simply as a function of volume or frequency for subsets of the data, to assess these relationships in the absence of interactions.

## Results

**Figure 2:**
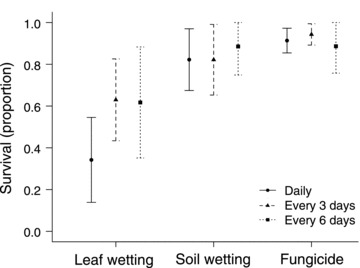
The proportion of seedlings surviving in leaf wetting, soil wetting, and fungicide methodologies when watered daily, every three days, and every six days. Error bars show one standard error.

Simulated rainfall frequency, volume, and method all significantly affected seedling survival, but the majority of the variation in survival was explained by the method of application (Table 1). Leaf wetting reduced survival to significantly lower levels than when leaf wetting was avoided or when fungicides were applied and although the effect of wetting method did not vary significantly with frequency, a significant frequency effect was only identified for the leaf wetting method (*F* = 9.68, df = 2,27, *P* < 0.001) when the data were analyzed separately for trays within each method as a function of frequency. Survival was significantly lower with daily leaf wetting, compared to leaf wetting every three or every six days, between which no significant difference was observed (Fig. 2). Watering frequency did not significantly affect survival in either the soil wetting or fungicide methodologies (Fig. 2).

**Table 1:** Analysis of deviance table showing the contribution of terms to the fitted model. Residual values are shown in parentheses. *P* values generated by *F* tests (with Type II sums of squares) comparing the significance of model terms: *P < 0.05; **P < 0.01; ***P < 0.001

Model term	df	Deviance	*P*
Volume	1 (76)	18.06 (281.29)	**
Frequency	2 (74)	21.50 (259.79)	**
Method	2 (72)	138.86 (120.93)	***
Volume × frequency	2 (70)	4.33 (116.60)	
Volume × method	2 (68)	0.42 (116.18)	
Frequency × method	4 (64)	15.58 (100.60)	
Volume × frequency × method	4 (60)	2.63 (97.97)	

**Figure 3:**
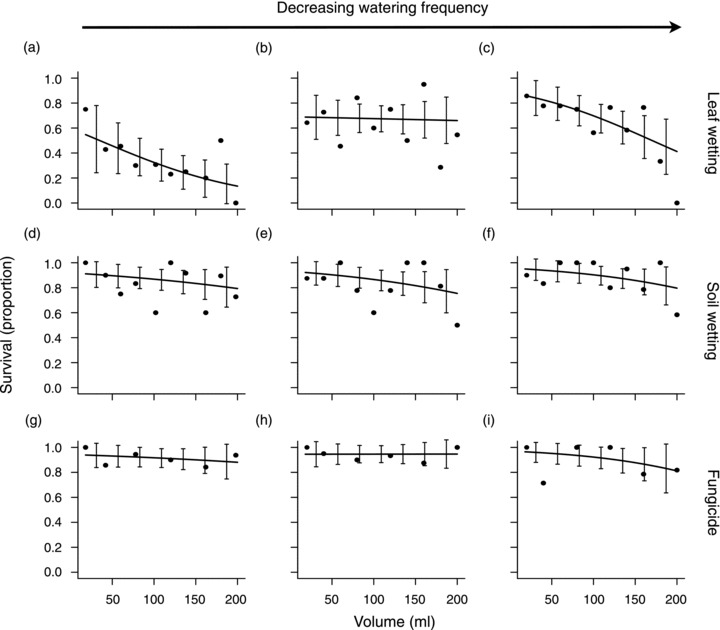
The proportion of seedlings surviving determined by the volume of simulated rainfall over a six-day period when rain was applied daily (a, d, and g), every three days (b, e, and h), and every six days (c, f, and i), under the leaf wetting (a–c), soil wetting (d–f), or fungicide (g–i) methodologies. Solid lines show the fitted relationship, while error bars show 95% confidence intervals.

Seedling survival was negatively affected by the total volume of water applied over the six-day period and none of the second- or third-order interactions including the volume term explained a significant proportion of the model deviance (Table 1). This suggests that the effect of volume did not differ with either watering frequency or method. However, when the relationship between survival and volume was analyzed individually for the trays within each frequency × method combination, a significant negative relationship was only identified when seedlings were watered daily or every six days, within the leaf wetting method (Fig. 3), indicating these data were responsible for the majority of this effect.

## Discussion

Our results suggest that altered rainfall regimes under climate change may have marked effects on plant mortality driven by fungal pathogens. Both watering frequency and volume were important determinants of pathogen-induced seedling mortality. Mortality was greatest with very regular (daily) watering, and declined significantly with less frequent watering, although no difference in mortality was observed between seedlings watered every three or every six days. This pattern was only observed in the leaf wetting method, while soil wetting did not produce significantly more mortality than the fungicide control. Furthermore, mortality increased with the volume of water and while this was observed across all methodologies, the effect was again far more pronounced under the leaf wetting method. The substantial increase in mortality in the leaf wetting method compared to both the soil wetting and fungicide methods implicates foliar fungal pathogens, which require frequent high volume rainfall events to transmit efficiently and drive mortality. Soil moisture alone did not have a significant effect on mortality, suggesting that any mortality driven by the soil biota were not responsive to variations in simulated rainfall. This contrasts with recent research, from Barro Colorado Island (Panama), implicating these organisms as primary determinants of the Janzen–Connell mechanism (Mangan et al. 2010). However, the natural enemies responsible for driving seedling mortality are likely to vary depending on host species and location, and while this study reveals the sensitivity of fungal pathogens to rainfall regimes, future studies should not ignore the potential for similar patterns to be driven by other natural enemies, such as bacteria and viruses.

If mortality from fungal pathogens is reduced through reduced rainfall, then the Janzen–Connell mechanism may be substantially less effective in maintaining diversity if tropical forests become drier, and if rainfall becomes more seasonal or erratic, as has been predicted (Malhi et al. 2008; Corlett 2011). This will be the case, particularly if patterns of density dependence are shifted toward weaker compensating forms (Freckleton and Lewis 2006; Bagchi et al. 2010) as a result of less frequent rainfall. The current experiment did not explicitly measure density dependence, but our previous work in the same shade house conditions has shown that *P. longicuspis* experiences overcompensating density-dependent mortality induced by fungal pathogens (Bagchi et al. 2010); seedlings in the previous study were watered daily and yet there was very little mortality at low density. This suggests that frequent, heavy rainfall is required to ensure that the establishment of individuals growing at high density is inhibited by fungal pathogens. However, this assumes that fungi will not evolve adaptations to altered climatic conditions (Singaravelan et al. 2008), which could maintain their capacity to drive seedling mortality. However, evidence that fungal pathogen activity is limited by low moisture levels in temperate forests (Gadgil 1974) suggests that the potential for adaptation is physiologically constrained. In addition, it should be noted that if the efficacy with which fungal pathogens drive seedling mortality is reduced in drier conditions, density-dependent seedling mortality might still occur if alternative natural enemies such as insect herbivores show increased activity. Future studies manipulating rainfall in the field should explore the potential for any corresponding change in the behavior of alternative natural enemies.

Existing research projecting biodiversity loss under climate change has largely focused on the ability of individual species to track changes in the distribution of their climate envelopes. This approach has been criticized for ignoring interspecific interactions such as those between plants and pathogens (Lavergne et al. 2010). While our results are limited to a single species they illustrate that the ecological processes thought to allow species coexistence, thereby maintaining diversity, are themselves likely to be highly sensitive to climate change. The Janzen–Connell mechanism is the leading theory explaining the coexistence of tropical forest trees but if climate change reduces its effectiveness this could contribute to the progressive erosion of diversity in topical forests.
